# Supplementary material for: Body mass index and critical care outcomes in hospitalized COVID-19 patients—A national cohort study
Source: PLoS One. 2025 Aug 19;20(8):e0329779. doi: 10.1371/journal.pone.0329779 (PMC12364376; doi:10.1371/journal.pone.0329779)
Supplement: Attachment — Submitted filename: review.docx [file pone.0329779.s001.docx]

Thank you for sharing the manuscript. Below is a detailed peer review of the article “Body Mass Index Impact on ICU Interventions and Outcomes in Hospitalized Patients with COVID-19 Infection – A National Population-Based Study.”

**Scientific Issues / Areas for Clarification:**

| Issue | Description | Recommendation |
| --- | --- | --- |
| Missing Ethics Detail | The ethics section says "N/A" yet claims IRB exemption later in the methods. | Clearly name the IRB, approval number, and exemption reason as per PLOS ONE guidelines. |
| Selection Bias | Exclusion of DNR/DNI patients and patients with certain comorbidities may introduce survivorship bias. | Discuss this as a limitation. |
| BMI Measurement Error | BMI in critical care can be inaccurate due to fluid shifts or supine positioning. | Address in limitations more explicitly. |
| Temporal Bias | The study uses 2020 data. The evolving nature of treatment protocols during that year may influence results. | Consider stratifying analysis by quarter/month if possible. |
| Effect Sizes | Although ORs are significant, many are modest (e.g., OR ~1.3). | Discuss clinical vs. statistical significance. |

**LANGUAGE & GRAMMAR**

**Common Issues Noted:**

1. **Verb Agreement & Sentence Structure:**
   - *Example*: “Our study revealed a higher utilization and duration of respiratory support in the ICU.” → Consider splitting or rephrasing.
   - *Correction*: “Our study revealed both higher utilization and longer duration of respiratory support in the ICU.”
2. **Repetitive Phrasing**:
   - Many parts redundantly explain the same physiological mechanisms (e.g., ACE-2 expression, cytokine storm).
   - Suggest condensing to improve readability.
3. **Overuse of “Obese patients were found…”**:
   - Consider varying language: e.g., "Individuals in the obese category showed...", "Participants with obesity demonstrated..."
4. **Inconsistent Use of Terms**:
   - Sometimes uses “COVID-19 infection” and other times just “COVID-19.” Recommend consistent usage.
5. **Ambiguous Wording**:
   - *Example*: “Patients classified as obese or overweight were noted to have extended stays.” → Use active voice: “Obese and overweight patients had longer ICU stays.”
6. **Citation Error**:
   - There is an “!!! INVALID CITATION !!!” placeholder under references.
   - Must be corrected before resubmission.
