# Supplementary material for: Body mass index and critical care outcomes in hospitalized COVID-19 patients—A national cohort study
Source: PLoS One. 2025 Aug 19;20(8):e0329779. doi: 10.1371/journal.pone.0329779 (PMC12364376; doi:10.1371/journal.pone.0329779)
Supplement: Attachment — Submitted filename: Comments.docx [file pone.0329779.s002.docx]

**Review Comments**

**1. Structure and Title**

The title is clear and effectively reflects the core content of the study. However, it could be slightly shortened for improved readability, for example:

"Body Mass Index and Critical Care Outcomes in Hospitalized COVID-19 Patients: A National Cohort Study"

Abstract: The abstract well-articulates the objectives, methodology, and key findings. However, in the Methods section, it would enhance clarity if the source of the data (e.g., specific database or registry) were explicitly mentioned.

**2. Introduction**

The introduction adequately sets the epidemiological and clinical context of the relationship between Body Mass Index (BMI) and respiratory disease severity, particularly in COVID-19 . However, more recent literature on the "obesity paradox" in respiratory illnesses could be referenced for a more comprehensive background

The hypothesis is clearly defined. Nevertheless, the significance of this study compared to previous work—such as the studies by Kompaniyets et al. (2021) and Kapoor et al. (2022) —should be more explicitly highlighted.

**3. Methods**

The study population and sample size (22,000 patients) are commendable, and data were drawn from the HCA Healthcare database, which is representative of 149 hospitals across 18 U.S. states. However, it would improve the methodological rigor if the authors clarified how variables such as prior vaccination status or previous SARS-CoV-2 infection (e.g., IgG serostatus) were accounted for in the analysis.

The BMI categories follow CDC standards, which is appropriate. However, the exclusion of the underweight group (BMI <18.5) from many analyses should be justified and discussed, especially if it was due to low sample size or confounding factors.

Statistical analysis: The use of ANOVA and regression models is suitable. However, further explanation should be provided regarding how confounding variables such as age, sex, and comorbidities (e.g., diabetes) were controlled in the models.

Limitations: The authors appropriately acknowledge limitations such as lack of access to detailed clinical data (e.g., inflammatory markers, medication use). However, this could be expanded by addressing how antiviral treatments and vaccination timing may have influenced outcomes, particularly during the pre-vaccination period.

**4. Results**

Table 3 is well-structured, but for the "Duration of Mechanical Ventilation" section, a bar chart would better emphasize differences than a tabular format.

Key Findings:

Obese patients had the highest rates of tracheostomy placement and prolonged mechanical ventilation, findings consistent with prior studies.

All-cause mortality did not show significant differences across BMI categories, which contrasts with some reported findings and deserves further discussion.

The multivariate analysis reported significant odds ratios for diabetes (OR: 1.70) , obesity (OR: 1.57), and chronic kidney disease (OR: 1.87) . However, the inverse association between age and tracheostomy need (OR: 0.98) requires clarification.

**5. Discussion**

The discussion compares well with previous studies such as Petrilli et al. (2020)

, but could benefit from referencing Simonnet et al. (2020), who demonstrated a strong association between obesity and mechanical ventilation requirements.

The clinical mechanisms linking obesity to mechanical ventilation needs are explained clearly. However, the role of altered leukocyte metabolism in obese individuals could be expanded upon to provide a deeper biological insight.

Limitations: The authors have appropriately addressed issues such as limited access to palliative care data and type of antiviral drugs used. Further elaboration on how obesity and associated comorbidities (e.g., diabetes) affected clinical decision-making and ICU management would strengthen this section.

Clinical Relevance: The results are well-connected to clinical practice, but the importance of weight management and obesity prevention strategies in reducing risks associated with COVID-19 severity should be emphasized.

**6. Conclusion**

The conclusions are well-summarized. To strengthen the impact, the authors could specify actionable strategies for managing obese patients with COVID-19, such as:

Increased vigilance in respiratory support.

Early decision-making protocols for tracheostomy when indicated.

Prioritizing vaccination and early intervention in obese patient groups.

**7. Tables and Figures**

Table 3 is well-constructed, but the variables "Need for Mechanical Ventilation" and "Tracheostomy Placement" would benefit from being presented as percentages to improve interpretability.

Figures 3 and 4 are appropriately structured, but the legends should clearly indicate the confidence intervals (CI) and beta/OR values for the regression outputs.

Figure Captions: The captions could be more precise. For instance, in Figure 4, it would improve clarity to specify the BMI range for the obese category.

**8. References**

The reference list includes recent and relevant sources. However, additional citations could be added for topics such as obesity and cytokine storm, and immunometabolism in obesity.

Some references, particularly

, should be updated to include the correct author names and publication years.
